# Diagnostic Value of CYFRA 21-1 in the Cerebrospinal Fluid for Leptomeningeal Metastasis

**DOI:** 10.1155/2017/2467870

**Published:** 2017-02-19

**Authors:** Zhen Zhang, Chenglin Tian, Qiang Shi, Jing Hao, Na Zhao, Zhijie Liu

**Affiliations:** ^1^Department of Neurology, PLA General Hospital, No. 28, Fuxing Road, Beijing 100853, China; ^2^Department of Neurology, PLA General Hospital, Hainan Branch, Haitang Bay, Sanya, Hainan 57200, China

## Abstract

Cerebrospinal fluid (CSF) cytology has low sensitivity for leptomeningeal metastasis (LM); thus, new markers are needed to improve the diagnostic accuracy of LM. We measured carcinoembryonic antigen (CEA) and cytokeratin 19 fragments (CYFRA 21-1) in paired samples of CSF and serum from patients with LM and patients with nonmalignant neurological diseases (NMNDs) as controls. Receiver operating curve analysis was performed to assess their diagnostic accuracy for LM. In patients with NMNDs, CEA and CYFRA 21-1 levels in the CSF were significantly lower than the serum levels. In patients with LM, there was no significant difference between the CSF and serum CEA levels, whereas the CYFRA 21-1 levels were significantly higher in the CSF than the serum. CSF/serum quotients of CYFRA 21-1 were higher than those of CEA in patients with LM and patients with NMNDs. CSF CYFRA 21-1 and CSF/serum quotient of CYFRA 21-1 had high accuracy for differentiating LM from NMNDs that was similar to CSF CEA and CSF/serum quotient of CYFRA 21-1, whereas serum CYFRA 21-1 is of poor diagnostic value. Measurement of CSF CYFRA 21-1 should not be overlooked in patients with suspected LM, even if the serum CYFRA 21-1 level is within normal limits.

## 1. Introduction

Leptomeningeal metastasis (LM) is the metastatic dissemination of malignant cells to the leptomeninges and subarachnoid space of the central nervous system. Cerebrospinal fluid (CSF) cytology remains the gold standard for the diagnosis of LM, but it has relatively low sensitivity [1]. The failure to demonstrate malignant cells in the CSF of patients with clinical signs and symptoms of LM remains a diagnostic challenge. Tumor markers (TMs) produced by malignant cells in the leptomeninges and subarachnoid space diffuse directly into the CSF. Thus, detecting elevated TMs in the CSF offers the possibility of an early diagnosis of LM. Indeed, the diagnostic value of the carcinoembryonic antigen (CEA) in the CSF for LM has been validated [2–8]. CA125, CA19-9, CA153, and cytokeratin 19 fragments (CYFRA 21-1) in CSF have also been reported to be useful indicators of LM [5, 7, 9]. In our institute, measurement of a panel of TMs in the CSF and serum has been employed to detect intracranial malignant metastasis. In clinical practice, “exceptionally” elevated CYFRA 21-1 in the CSF have been observed in some cases of LM, indicating that the CYFRA 21-1 level in the CSF does not generally correspond with that in the serum. This phenomenon suggests that the diagnostic value of CYFRA 21-1 in the CSF for LM might be different from that in the serum for primary tumor and/or systematic metastasis. In the present study, we retrospectively evaluated the changes in CEA and CYFRA 21-1 in paired samples of CSF and serum in patients with LM arising from systemic solid tumors. The diagnostic accuracy of CEA and CYFRA 21-1 for LM was evaluated through comparison to patients without malignant diseases.

## 2. Material and Methods

### 2.1. Assay of CEA and CYFRA 21-1

CEA and CYFRA 21-1 levels in the CSF and serum were measured with an immunochemiluminescent assay on a Roche MODULAR ANALYTICS E170 analyzer. Specifically, the serum or CSF sample, biotinylated monoclonal CEA-specific or CYFRA 21-1-specific antibody, and a monoclonal CEA-specific or CYFRA 21-1-specific antibody (Elecsys CEA and CYFRA 21-1 immunochemiluminescent assay kit) labeled with a ruthenium complex were reacted to form a sandwich complex. After addition of streptavidin-coated microparticles, the complex bound to the solid phase via the interaction of biotin and streptavidin. The microparticles were then magnetically captured onto the surface of the electrode, and unbound substances were removed. Application of voltage to the electrode then induced chemiluminescent emission, which was measured by a photomultiplier. The results were determined via reference to a calibration curve that was instrument-specifically generated. The lower detection limits for CEA and CYFRA 21-1 were 0.2 *μ*g/mL and 0.1 ng/mL, respectively. Measurement of a panel of TMs, including CEA and CYFRA 21-1, is a routine aspect of the CSF analysis workup for ascertaining the diagnosis of intracranial malignant metastasis in our institution and had been approved by the institutional ethics committee.

### 2.2. Patients

#### 2.2.1. Patients with LM

The data of patients with LM were retrospectively collected among patients admitted to our hospital from 2005 to 2015. The diagnosis of LM in this study was based on at least one of the following criteria: (1) CSF cytology identified malignant cells; (2) if CSF cytology was negative for malignant cells, primary tumor and/or systematic metastasis was ascertained by pathologic evidence obtained through biopsy or tumor resection, and the clinical manifestation, neuroimaging characteristics, and results of the routine CSF analysis workup were consistent with those of LM [10–14]. Patients fulfilling the above diagnostic criteria and undergoing combined measurements of CEA and CYFRA 21-1 in paired samples of CSF and serum were ultimately included in this study.

#### 2.2.2. Patients with Nonmalignant Neurological Diseases (NMNDs)

For each patient with LM, the data of a control patient with NMND were retrospectively reviewed. As of January 1, 2011, patients who met the following criteria were finally included in the control group: (1) age and sex were matched with the corresponding patient with LM; (2) a lumbar puncture had been performed; (3) CEA and CYFRA 21-1 were measured in paired samples of the CSF and serum for diagnostic purpose (the two samples had to have been collected from the patient within one week); (4) central nervous system and systematic malignant disease had been ruled out.

### 2.3. Statistical Analysis

CEA and CYFRA 21-1 levels in paired samples of the CSF and serum were retrospectively evaluated in patients with LM and in control patients with NMNDs. The Wilcoxon signed ranks test was employed to compare the CSF level with the serum level. CSF/serum quotients of CEA (*Q*_CEA_) and CYFRA 21-1 (*Q*_CYFRA  21-1_) were calculated. When CSF CEA was below the lower detection limit (0.2 *μ*g/mL), 0.2 *μ*g/mL was used as CSF level in calculating *Q*_CEA_.   *Q*_CEA_ was compared with *Q*_CYFRA  21-1_ in patients with LM and patients with NMNDs, respectively, by Wilcoxon signed ranks test. We used receiver operating characteristic (ROC) analysis to determine the area under the curve to assess the diagnostic accuracy of CEA and CYFRA 21-1 in CSF, CEA and CYFRA 21-1 in serum, *Q*_CEA_ and *Q*_CYFRA  21-1_ for LM arising from systemic solid tumors. We included diagnostic accuracy measures of optimal cut-off values based on ROC analysis by maximizing the Youden index. All statistical analyses were performed using the software SPSS 19.0 (Chicago, IL, USA).

## 3. Results

Sixty patients with LM (28 males and 32 females with an average age of 52.45 years, range: 40–77 years, standard deviation: 8.67 years) were included in this study. The primary tumor arose from the lung in 53 patients, stomach in three patients, and colon, liver, breast, and pleura in one patient each. The pathological type of the primary tumor was determined to be adenocarcinoma in 54 patients and mesothelioma in one patient through CSF cytology and/or pathological analysis of the primary tumor. In the remaining five patients, the CSF cytology assay only revealed malignant cells in the CSF; however, the exact pathological type could not be clarified and pathological evidence of the primary tumor was not available.

In the LM patients, there was no significant difference between the CSF and serum level of CEA (*P* = 0.54), whereas the CSF level of CYFRA 21-1 was significantly higher than its serum level (*P* < 0.01) (Table 1). Two patients with LM had CSF CEA level below 0.2 *μ*g/mL, the lower detection limit. CSF CYFRA 21-1 was above the lower detection limit in all patients.

In patients with NMNDs, the levels of both CEA and CYFRA 21-1 in the CSF were extremely low and were significantly lower than those in the serum (Table 1). Only one patient had CSF CEA level above the lower detection limit. CSF CYFRA 21-1 was above the lower detection limit in all patients.

Except a 51-year-old man with virus encephalitis whose *Q*_CYFRA  21-1_ is 1.19, *Q*_CEA_ and *Q*_CYFRA  21-1_ were below 1.0 in all other patients with NMDs, and *Q*_CYFRA  21-1_ was significantly higher than *Q*_CEA_ in both patients with LM and patients with NMNDs (Table 2).

**Figure 1:**
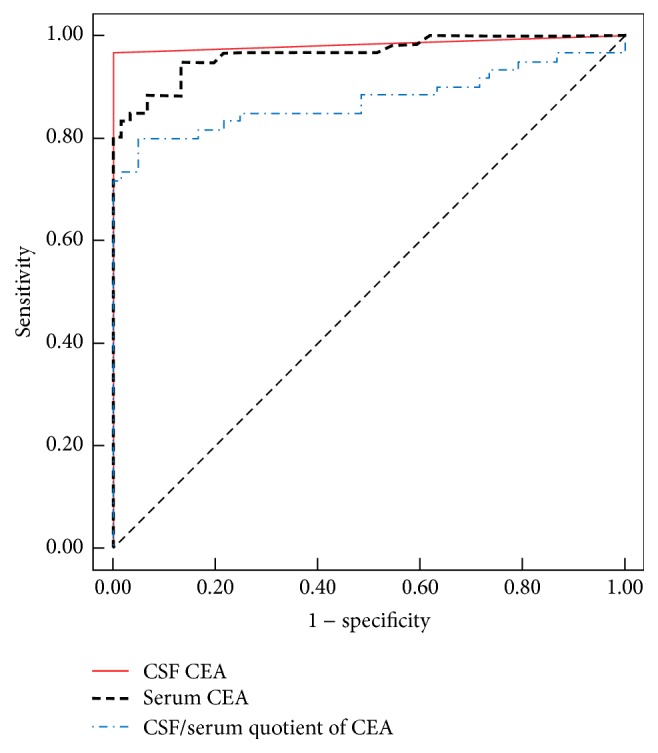
Receiver operating characteristic curves of cerebrospinal fluid (CSF) carcinoembryonic antigen (CEA), serum CEA, and CSF/serum quotient of CEA for differentiating between leptomeningeal metastasis and nonmalignant neurological diseases.

**Figure 2:**
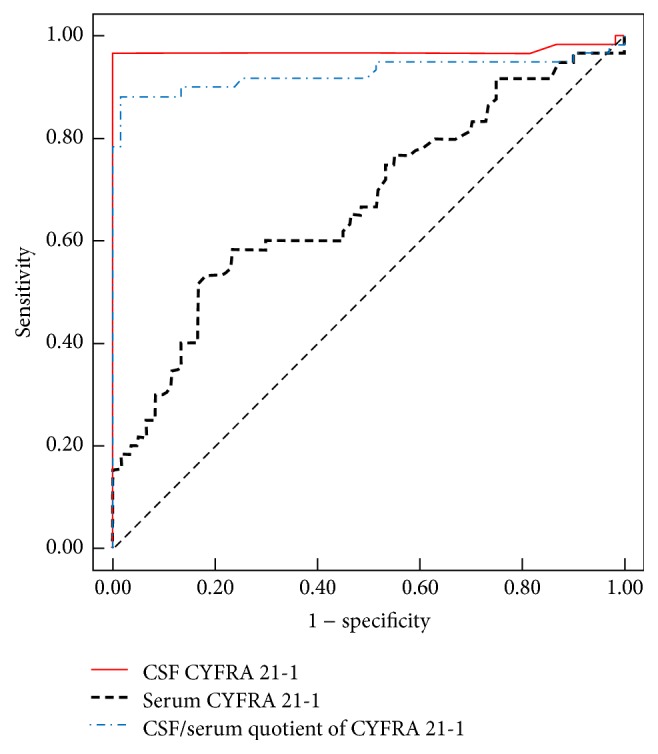
Receiver operating characteristic curves of cerebrospinal fluid (CSF) CYFRA 21-1, serum CYFRA 21-1, and CSF/serum quotient of CYFRA 21-1 for differentiating between leptomeningeal metastasis and nonmalignant neurological diseases.

ROC analysis demonstrated the high diagnostic accuracy of CSF CEA, CSF CYFRA 21-1, serum CEA, *Q*_CYFRA  21-1_, and *Q*_CEA_ for the diagnosis of LM arising from a systemic solid tumor. However, the serum CYFRA 21-1 diagnostic performance was poor, which mainly resulted from its low sensitivity (Figures 1 and 2, Table 2). The ROC was used to determine the cut-off value to separate patients with LM with solid tumors from those with NMNDs, which was 0.279 *μ*g/mL for CSF CEA, 4.115 *μ*g/mL for serum CEA, 1.145 ng/mL for CSF CYFRA 21-1, 2.61 ng/mL for serum CYFRA 21-1, 0.45 for *Q*_CEA_  , and 0.85 for *Q*_CYFRA  21-1_. The diagnostic accuracy of CEA in the CSF and serum, CYFRA 21-1 in the CSF and serum, *Q*_CEA_  , and *Q*_CYFRA  21-1_ at the optimal cut-off values is summarized in Table 3.

## 4. Discussion

In our patients, CSF and serum CEA both showed high sensitivity and specificity for the diagnosis of LM when differentiating from the control group of patients with NMNDs. There was no significant difference between the CSF and serum levels of CEA. However, in contrast to the estimated differences of CEA in the CSF and serum, the CYFRA 21-1 level in the CSF was significantly higher than that in the serum. Thus, CSF CYFRA 21-1 shows very high sensitivity and specificity for the diagnosis of LM, which is similar to that of CSF CEA. By contrast, the sensitivity of serum CYFRA 21-1 was only half that of serum CEA. Since patients with NMNDs were used as controls in the present study, diagnostic performance of serum CEA and CYFRA 21-1 actually reflected their value in differentiating primary systemic tumor from NMND rather than that in differentiating LM from systemic solid tumor without LM. Different optimal cut-off values of serum CYFRA 21-1 with sensitivity from 66.1% to 89.3% for non-small cell lung cancer (NSCLC) were reported according to the control group patients with different benign pulmonary disease. The optimal cut-off value to different LM from NMNDs determined in this study was almost identical to that for NSCLC when patients with pulmonary tuberculosis were used as control group [15]. The relative low sensitivity of serum CYFRA 21-1 in our patients might be attributed to primary tumors consisting of different pathological type. Another exception finding in this study is that  *Q*_CYFRA  21-1_ larger than 1.0 was found in one patient with NMND. We presumed that this paradoxical change might be caused by assay bias or that CSF and serum sample were not collected on the same day.

Intrathecal fraction is more accurate for detecting abnormal intrathecal synthesis of biomarkers [16] and has been employed in some previous related reports [5, 8]. However, our data were retrospectively collected and CSF albumin was not available in most cases. CSF *Q*_CEA_ and *Q*_CYFRA  21-1_ were used to reflect their intrathecal synthesis and both showed high sensitivity and specificity for the diagnosis of LM. We also found that *Q*_CYFRA  21-1_ was higher than *Q*_CEA_ in both patients with LM and patients with NMNDs. The molecular weight of CYFRA 21-1 is 30 kDa, whereas that of CEA is 180 kDa; thus, the trans-blood-brain-barrier (BBB) diffusion of CYFRA 21-1 should be more active than that of CEA. In other words, it should be easier to balance CYFRA 21-1 levels in the CSF and serum with all else being equal. So, in patients with NMNDS, higher *Q*_CYFRA  21-1_ most likely results from more active trans-BBB diffusion of CYFRA 21-1 from blood to CSF. Considering that the CSF CYFRA 21-1 level was higher than the serum CYFRA 21-1 in most patient, higher *Q*_CYFRA  21-1_ cannot be attributed to trans-BBB diffusion in patients with LM. Higher *Q*_CYFRA  21-1_ suggested other underlying mechanisms for elevation of the CSF CYFRA 21-1 level that is strong enough to counterbalance the lowering effect from trans-BBB diffusion.

One possible mechanism is that tumor cells metastasizing to the leptomeninges synthesize and secrete CYFRA 21-1 at higher levels than their counterparts in the primary tumor or systemic metastasis. That is, during the process of metastasis, tumor cells might acquire the capability or increase their inherent capability to synthesize CYFRA 21-1, without a corresponding change in the synthesis ability for CEA. According to the “seed and soil” hypothesis, metastatic colonization of a given organ is not due to chance, but is influenced by a specific affinity of certain tumor cells (the “seed”) for the milieu of certain organs (the “soil”) [14]. Central nervous system metastasis is a complex process involving the interaction between the metastatic tumor cells and the central nervous system microenvironment [17, 18]. For example, astrocytes have been demonstrated to induce brain-metastatic breast cancer cells expressing matrix metalloproteinase via paracrine signaling [14]. Neurotrophins seem to promote melanoma cell invasion by stimulating and sustaining growth and migration, for example, by inducing the expression of heparanase or cytoskeletal rearrangements [14]. In a case of LM from lobular breast cancer, the malignant cells in CSF lost their hormone receptors expressed by the primary tumor [19]. In addition to the interaction between the central nervous system microenvironment and tumor cells after they reach the central nervous system, clone selection in the formation of central nervous system metastasis might also result in molecular characteristics of tumor cells in LM that are distinct from those in the primary tumor. Upregulated expression of cytokeratin-19 (CK-19), the parent protein of CYFRA 21-1, has proven to be an independent risk factor of intraliver and lymph node metastasis in hepatocellular carcinoma [20, 21]. An in vitro study also demonstrated that overexpression of CK-19 in hepatocellular carcinoma cells was associated with increased metastatic behavior [20]. In low-grade endometrioid adenocarcinoma, expression of CK-19 is restricted to the central areas of the larger conventional neoplastic glands and to the infiltrative tumor components [22]. If tumor cells with overexpression of CK-19 also have great potential to invade the central nervous system, the proportion of CK-19-positive tumor cells in LM will be higher than those in the primary site or systemic metastasis, thus resulting in a greater amplitude of elevation of CYFRA 21-1 in the CSF than in the blood. Upregulation of CK-19 expression facilitated by the central nervous system microenvironment after the tumor cells reach leptomeninges also cannot be ruled out as an alternative mechanism.

Clearance of TMs also affects their levels in the body fluid compartment. Clearance of TMs in the CSF is mainly determined by their speed of diffusion across the BBB to the blood. These markers might have a similar clearance speed from the CSF, as they are all large molecules that cannot easily cross the BBB. However, in actuality, the half-life of different TMs in the blood varies greatly. In patients with malignant lung neoplasms, the half-life of CEA was reported to be 1.3 days after curative resection, whereas that of CYFRA 21-1 was found to be much faster at 0.17 days [23]. This might explain, at least in part, the findings of the present study.

Our study has some limitations worth mentioning. First, the optimal control patients for assessing the diagnostic accuracy of TMs in the CSF for LM are those with pathologically matched solid tumors without central nervous system metastasis. Nonetheless, lumbar puncture is unnecessary in patients without suspected central nervous system metastasis and it is ethically infeasible to perform lumbar puncture just for study purpose. Second, intrathecal fraction is usually employed in evaluating biomarkers in CSF for diagnostic purpose. It reflects intrathecal synthesis of biomarker more accurately than CSF concentration by adjusting the effect of BBB dysfunction. In LM, BBB is usually damaged, elevated TM in CSF may result from trans-BBB diffusion from blood rather than intrathecal synthesis. Because of the retrospective nature of our study, intrathecal fraction could not be obtained. Nonetheless, this limitation does not weaken our findings about the diagnostic value of CSF CYFRA 21-1 for LM. CSF CYFRA 21-1 was higher than serum in most patients with LM, which rule out the possibility that elevated CSF CYFRA 21-1 solely resulted from trans-BBB diffusion from blood. Third, 0.2 *μ*g/mL was used to calculate *Q*_CEA_ in patients whose CSF CEA is below the lower detection limit. This limitation resulted in artificially high values CSF CEA and *Q*_CEA_. However, we believe that this limitation does not weaken the diagnostic value of CSF CEA for LM. Even artificially elevated CSF values of CEA and *Q*_CEA_S in 59 NMND patients were used as controls, CSF CEA (artificially elevated in only 2 LM patients) was still demonstrated to be high sensitive and specific for LM. In other words, the actual sensitivity and specificity of CSF CEA and *Q*_CEA_ for LM may be even higher than the results of our study. Fourth, the LM in this group of patients consisted of different pathological types and the number of patients was relatively small. Our findings are most likely relevant for the major pathological types such as lung adenocarcinoma; however, whether or not they are applicable to the more relatively rare pathological type requires further verification.

Notwithstanding the above limitations, from a clinical perspective, our findings highlight the diagnostic significance of CSF CYFRA 21-1 as a marker for LM. Therefore, CYFRA 21-1 in the CSF should not be overlooked in patients with suspected LM, even if their serum CYFRA 21-1 levels are within normal limits.

## Figures and Tables

**Table 1:** Comparison of CSF and serum levels of CEA and CYFRA 21-1 in patients with leptomeningeal metastasis and patients with nonmalignant neurological diseases.

Patients	Tumor markers	Sample	Minimum	25% percentile	Median	75% percentile	Maximum	*P* value
LM	CEA	CSF	≤0.20	4.97	30.74	129.29	2105.00	0.20
	(*μ*g/mL)	Serum	1.29	6.56	17.18	86.76	1118.00	
	CYFRA 21-1	CSF	0.50	3.24	6.95	25.73	1180.00	0.00
	(ng/mL)	Serum	0.38	1.81	2.61	4.10	126.20	

NMNDs	CEA	CSF	≤0.20	≤0.20	≤0.20	≤0.20	0.23	0.00
	(*μ*g/mL)	Serum	0.45	1.02	1.52	2.08	4.89	
	CYFRA 21-1	CSF	0.40	0.66	0.74	0.79	1.01	0.00
	(ng/mL)	Serum	0.85	1.31	1.95	2.36	6.08	

LM: leptomeningeal metastasis, NMNDs: nonmalignant neurological diseases.

**Table 2:** CSF/serum quotient of CEA and CYFRA 21-1 in patients with leptomeningeal metastasis and patients with nonmalignant neurological diseases.

Patients	CSF/serum quotient	Minimum	25% percentile	Median	75% percentile	Maximum	*P*
LM	CEA	0.03	0.36	0.96	3.20	31.88	0.00
	CYFRA 21-1	0.03	1.33	2.81	10.20	350.15	

NMNDs	CEA	0.04	0.10	0.13	0.30	0.44	0.00
	CYFRA 21-1	0.10	0.30	0.39	0.57	1.19	

LM: leptomeningeal metastasis, NMNDs: nonmalignant neurological diseases.

**Table 3:** Diagnostic performance of CEA and CYFRA 21-1 in the CSF, CEA, and CYFRA 21-1 in the serum and CSF/serum quotient of CEA and CYFRA 21-1 for differentiating leptomeningeal metastasis from nonmalignant neurological diseases.

Tumor markers		AUC (95% CI)	Sensitivity (95% CI)	Specificity (95% CI)
CEA	CSF	0.98 (0.96–1.00); *P* < 0.01	0.97 (0.88–0.99)	1.00 (0.93–1.00)
	Serum	0.97 (0.94–1.00); *P* < 0.01	0.86 (0.74–0.94)	0.97 (0.87–0.99)
	CSF/serum quotient	0.87 (0.80-0.95); *P* < 0.01	0.72 (0.58–0.82)	0.970 (0.79–0.96)

CYFRA 21-1	CSF	0.97 (0.93–1.00); *P* < 0.01	0.97 (0.88–0.99)	1.00 (0.93–1.00)
	Serum	0.67 (0.58–0.77); *P* < 0.01	0.50 (0.41–0.59)	0.83 (0.71–0.91)
	CSF/serum quotient	0.93 (0.87–0.99); *P* < 0.01	0.88 (0.77–0.95)	0.97 (0.87–0.99)
